# Creative music therapy to promote brain function and brain structure in preterm infants: A randomized controlled pilot study

**DOI:** 10.1016/j.nicl.2020.102171

**Published:** 2020-01-13

**Authors:** Friederike Barbara Haslbeck, Andras Jakab, Ulrike Held, Dirk Bassler, Hans-Ulrich Bucher, Cornelia Hagmann

**Affiliations:** a Department of Neonatology, University Hospital Zurich and University Zurich, Frauenklinikstrasse 10, 8091 Zürich, Switzerland; b MR Research Center, University Children's Hospital Zurich, Steinwiesstrasse 75, 8032 Zürich, Switzerland; c Department of Biostatistics Epidemiology, Biostatistics and Prevention Institute UZH, Hirschengraben 84, 8001 Zürich, Switzerland; d Department of Neonatology and Pediatric Intensive Care, Children's University Hospital of Zurich, Steinwiesstrasse 75, 8032 Zürich, Switzerland; e Children's Research Center, University Children's Hospital Zurich, Steinwiesstrasse 75, 8032 Zürich, Switzerland

**Keywords:** Preterm infants, Music therapy, Resting state fMRI, Functional connectivity, Thalamocortical processing, Neurodevelopment, AAL, Automated Anatomical Labeling, CMT, creative music therapy, DT-MRI, diffusion tensor magnetic resonance imaging, EPI, echo-planar imaging, FA, fractional anisotropy, FD, framewise displacement, FDR, false discovery rate, fMRI, functional magnetic resonance imaging, ICA, independent component analysis, NICU, neonatal intensive care unit, NIRS, near infrared spectroscopy, ODF, orientation distribution function, PMCC, product moment correlation coefficient, ROI, region of interest, rsfMRI, resting-state functional magnetic resonance imaging, SC, structural connectivity strength, TBSS, tract-based spatial statistics, TCLP, thalamocortical lag projection, TE, echo time, TFCE, threshold-free cluster enhancement, TR, repetition time

## Abstract

Cognitive and neurobehavioral problems are among the most severe adverse outcomes in very preterm infants. Such neurodevelopmental impairments may be mitigated through nonpharmacological interventions such as creative music therapy (CMT), an interactive, resource- and needs-oriented approach that provides individual social contact and musical stimulation. The aim was to test the feasibility of a study investigating the role of CMT and to measure the short- and medium-term effects of CMT on structural and functional brain connectivity with MRI. In this randomized, controlled clinical pilot feasibility trial, 82 infants were randomized to either CMT or standard care. A specially trained music therapist provided CMT via infant-directed humming and singing in lullaby style. To test the short-term effects of CMT on brain structure and function, diffusion tensor imaging data and resting-state functional imaging data were acquired. Clinical feasibility was achieved despite moderate parental refusal mainly in the control group after randomization. 40 infants remained as final cohort for the MRI analysis. Structural brain connectivity appears to be moderately affected by CMT, structural connectomic analysis revealed increased integration in the posterior cingulate cortex only. Lagged resting-state MRI analysis showed lower thalamocortical processing delay, stronger functional networks, and higher functional integration in predominantly left prefrontal, supplementary motor, and inferior temporal brain regions in infants treated with CMT. This trial provides unique evidence that CMT has beneficial effects on functional brain activity and connectivity in networks underlying higher-order cognitive, socio-emotional, and motor functions in preterm infants. Our results indicate the potential of CMT to improve long-term neurodevelopmental outcomes in children born very preterm.

## Highlights

• We tested whether CMT affects neurodevelopment in preterm infants.
• CMT improved functional brain networks and functional brain integration.
• CMT improved thalamocortical processing.
• Improvement in prefrontal, supplementary motor and temporal brain regions.
• Association with higher-order cognitive, socio-emotional, and motor functions.

## 1 Introduction

Very preterm birth is associated with a higher prevalence of neurodevelopmental impairments. Indeed, preterm birth is a risk factor for the development of cognitive and neurobehavioral problems that lead to academic underachievement (Twilhaar et al., 2018; Aarnoudse-Moens et al., 2009) and psychiatric disorders (Johnson et al., 2010). Among the most common adverse outcomes are deficits in executive functions and behavioral problems (Twilhaar et al., 2018). These impairments persist from early childhood into adolescence and adulthood (Nosarti et al., 2007; Wehrle et al., 2018).

Socio-emotional and auditory deprivation and the stressful environment in the neonatal intensive care unit (NICU) may negatively impact brain maturation (Hodel, 2018). Separation from mothers can increase very preterm infants’ stress response (Udry-Jørgensen et al., 2011) and may adversely affect neurodevelopment, as demonstrated in animal studies (Champagne and Curley, 2009; Weaver et al., 2004). Very preterm infants are not exposed to the nurturing intrauterine vibro-acoustic experience, characterized by the deep regular rhythms of the maternal heartbeat and the mother's voice in melody and prosody (Moon et al., 2013). In contrast, preterm birth exposes the developing brain to various harmful stimuli during the time in neonatal intensive care, both biological, such as infection, hypoxia, ischaemia (Khwaja and Volpe, 2008), and environmental, such as high-frequency, random noise from machines, light, and pain. Very preterm infants are particularly sensitive to these stressors because their brains are undergoing the most sensitive and rapid period of synaptic development (Kostovic and Vasung, 2009).

Auditory experience influences early brain development, as has been demonstrated in human and animal studies (Dahmen and King, 2007; de Villers-Sidani et al., 2008), and musical learning starts before birth (Perani et al., 2010; Huotilainen and Näätänen, 2010). Music perception can activate various limbic and paralimbic structures and improve network connectivity in children and adults (Reybrouck et al., 2018). Music modulates synaptic plasticity and promotes neurobiological processes, neuronal learning, and readjustment in the human and animal brain (Rickard et al., 2005; Fujioka et al., 2006).

The extent and severity of neurodevelopmental impairment in preterm infants due to their adverse sensory-social experience may be mitigated through creative music therapy (CMT), which encompasses nurturing social contact and musical stimulation. CMT is an individualized, interactive, resource- and needs-oriented approach (Haslbeck, 2014; Haslbeck and Hugoson, 2017). CMT emphasizes that being responsive to music is an intrinsic quality of the human being, no matter how ill, disabled, or premature an infant may be (Nordoff and Robbins, 2012). CMT therapists assess the infant's breathing patterns, facial expressions, and gesticulations to construct a musical entrained response with humming and singing in lullaby style. Furthermore, CMT is a family-integrating music therapy approach, and the parents are involved individually in the therapeutic process, for example by providing music therapy during skin-to-skin contact to foster an intuitive parent–infant interaction and so to strengthen the bonding process.

Several studies have shown positive short-term music therapy outcomes in arousal, behavior, and respiratory rate (Haslbeck, 2012; Hartling et al., 2009; Standley, 2012). A multisite trial (Loewy et al., 2013) and a recent meta-analysis (Bieleninik et al., 2016) have confirmed these favorable effects, particularly the beneficial effect of music therapy on respiratory rate in preterm infants and on maternal anxiety. By contrast, neurodevelopmental short- and mid-term outcomes have rarely been evaluated.

To address this gap, a prospective randomized controlled pilot trial examining the short- and mid-term neurodevelopmental and neurobehavioral outcomes in preterm infants was conducted with CMT (Haslbeck et al., 2017; Haslbeck and Bassler, 2018). We hypothesized that CMT leads to a better neurodevelopmental trajectory that may also manifest in structural and functional changes in the developing brain. Neuroimaging by means of diffusion tensor MRI (DTI) and resting-state functional MRI (rsfMRI) has the potential not only to detect the changes in brain development related to preterm birth (Dubois et al., 2014; Rogers et al., 2018) and the relationship between brain connectivity and neurodevelopmental sequalae (Rogers et al., 2018; Parikh, 2016) but also to demonstrate the neuroprotective effects of interventional studies on brain development (O'Gorman et al., 2015; Jakab et al., 2019).

Therefore, the aim of our work was to measure the short- to medium-term effects of CMT on the structural, connectivity, and functional development of the newborn brain with noninvasive MRI techniques. We hypothesized that tract-based spatial statistics of DTI data would be the optimal choice of method to reveal possible medium-term effects of CMT on structural brain development, axonal development, and myelination, while short-term effects would more likely result in subtle changes in the functional connectivity structure. Further aims were to test clinical, recruitment, and outcome measurement feasibility.

## 2 Materials and methods

The study received approval from the Ethics Committee of Canton Zurich, Switzerland (KEK-ZH 2014-0655) and from the Swiss Agency for Therapeutic Products (Swissmedic), Audit No.: CTCQA14. The trial is registered at ClinicalTrials.gov (Number: NCT02434224. The study is designed as a prospective, single-center, between-subject randomized, clinical pilot trial in preparation for a full-scale trial (Haslbeck et al., 2017).

### 2.1 Study feasibility

The success of recruitment was measured by summarizing randomization rates and reasons for withdrawal compared to available patients listed in the screening log. The levels of missing outcome measures and loss to follow-up rates were generated at the end of the study and will serve to calculate the sample size power for the planned multicenter trial.

### 2.2 Participants

Eighty-two preterm infants were recruited on NICU at the University Hospital Zurich. Inclusion criteria were gestational age at birth < 32 weeks, chronological age ≥ 7 days of life at start of the intervention, clinically stable, and parental informed consent obtained. Exclusion criteria were genetically defined syndrome, congenital malformation adversely affecting life expectancy or neurodevelopment, high-grade intraventricular hemorrhage, and/or cystic white matter lesions. In addition, those infants admitted for palliative care were excluded from the study.

### 2.3 Randomization

Following parental signed informed consent, randomization was performed using a computer-generated list created before study initiation. Allocation ratio was 1:1. Allocation concealment and random sequence minimized selection bias. Sealed, opaque, numbered envelopes were opened only after the envelope had been irreversibly assigned to the participant. The allocation process was monitored by the study sponsor to preserve concealment. Very preterm infants randomized to the control group received standard care including skin-to-skin care with the parents as delivered in the NICU at the University Hospital of Zurich. Very preterm infants allocated to the CMT group received CMT additionally to the standard care as explained below.

### 2.4 Intervention

A well-trained and experienced music therapist (HF) formulated individualized, culturally adapted treatment plans based on an initial child–parent assessment, which included assessment of parental needs, musical heritage, culture, context, and parental integration in the therapeutic process. The therapist individually adapted the aims over the course of hospitalization in accordance with the principles of neonatal music therapy and family-integrating care approaches (Haslbeck, 2014; Haslbeck and Hugoson, 2017; O'Brien K et al., 2018). Therapy sessions started immediately following parental consent, two to three times per week in the morning after feeding time. Each CMT intervention lasted approximately 20 min and was directed to the infant at the bedside alone or with the parents in skin-to-skin contact. Each infant received a minimum of eight sessions of CMT, since this is the recommended number of sessions suggested to measure a therapeutic effect (Hanson-Abromeit et al., 2008). During the CMT session, the infant was lying in the incubator or in warmers. Mostly, when the infants tolerated touch, the session started with an initial touch, for instance of the head and feet, which was transformed into therapeutic touch to offer contact and to feel and stimulate the breathing rhythm of the infant (Haslbeck, 2014; Hanley, 2008). After an initial period of observation, the humming was faded in smoothly, starting with some long, calm notes developing over time into a smooth melody in lullaby style. The humming and singing were individually tailored to the breathing rhythm, facial expression, and gesture of the infant. The families’ musical heritage and culture were addressed by integrating musical preferences into the improvisation, for example by incorporating the parents’ favorite song (e.g., folk song of their culture, pop song) in lullaby style (Loewy et al., 2013). At the end of the session, the music faded out smoothly, and the therapist cautiously removed her hands.

**Fig. 1:**
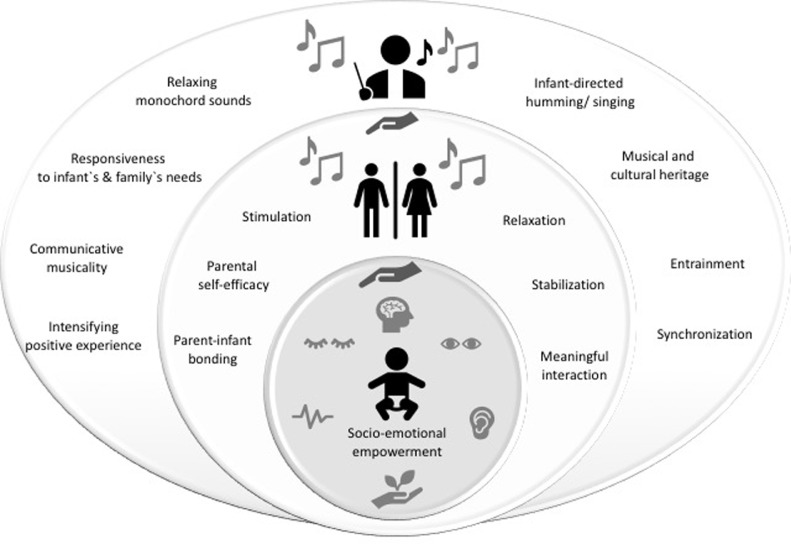
*Creative Music Therapy with preterm infants and their parents*. Responsiveness, communicative musicality, and empowerment via infant-directed humming/ singing and relaxing monochord sounds.

During therapy with the parents, the humming and singing was provided in the same manner, but the infant was placed on the parent's chest in skin-to-skin contact or, when the infants were older, on the parent's lap. Additionally, a vibro-acoustic monochord^2^ was used to accompany the singing and to fade the music in and out. This was placed at the elbow of the parents to allow its vibrations to transmit relaxation, particularly to parents still in a post-traumatic state. The parents were invited to relax, to observe their infant, or to sing along with the music therapist and were empowered to hum to their infant in general. If appropriate, the music therapist shared her perceptions of the infant's behavioral state and reactions (e.g., smiling, finger movements, if these occurred) to encourage positive and sensitive parent–infant interactions to empower the parents and parent–infant attachment. Before hospital discharge, the music therapist provided a final consultation and debriefing discussion with the parents, including therapeutic recommendations and offering music consultation for the first year of life (Fig. 1) (Haslbeck et al., 2017).

### 2.5 MRI acquisition

MRI was acquired during natural sleep. The infants were protected against scanner noise by ear plugs (attenuation: 24 dB; Earsoft; Aearo) and Minimuffs (Attenuation: 7 dB; Natus) (O'Gorman et al., 2015). The heart rate and oxygen saturation of very preterm infants were monitored during imaging.

All imaging was acquired using a 3.0T Siemens Scanner at the University Hospital of Zurich equipped with an eight-channel phased array head coil. DTI was acquired with a pulsed gradient spin echo planar (EPI) sequence with TE = 92 ms, TR = 5 s, flip angle = 90°, field of view = 18 cm, matrix = 128 × 128, slice thickness = 3 mm, 60 non-collinear gradient encoding directions with *b* = 650 s/mm² and two interleaved *b* = 0 images. Resting-state fMRI (fMRI) was acquired with a T2*-weighted EPI sequence, TE = 30 ms, TR = 2 s, flip angle = 69°, field of view=18 cm, matrix = 64 × 64, voxel size = 3 × 3 mm, slice thickness = 3 mm, slice gap = 0.3 mm. Structural MRI was performed using T2-weighted turbo spin echo sequence, TE = 106 ms, TR = 3.6 s, flip angle = 135°, field of view = 18 cm, matrix = 448 × 235, voxel size = 0.4 × 0.4 mm, slice thickness = 2.5 mm, slice gap = 0.25 mm.

#### 2.5.1 Diffusion tensor MR image preprocessing

DTI data were processed using a script that combines the various functionalities of FMRIB Software Library software FSL. Masking of the brain in the DTI images was done using the brain extraction tool in FSL with the initial brain radius set to 35 mm. Masking quality was checked visually, and the mask was manually corrected upon anatomical inconsistency. We used the CUDA 8.0 implementation of the EDDY (Andersson and Sotiropoulos, 2016) in FSL 6.0 to correct for eddy-current-induced distortions and head movements. Slice-to-volume reconstruction with outlier detection was part of the processing (Andersson et al., 2016), which was previously demonstrated to tackle artefacts emerging from head movements. The parameters used in the eddy correction step are detailed in the code of the postprocessing script (*will be shared as Digital Supplement*).

#### 2.5.2 Diffusion tensor MR image analysis: tract-based spatial statistics

Tract-based spatial statistics (TBSS 1.2) was performed using the pipeline previously reported (Smith et al., 2006). We used the fractional anisotropy (FA) images generated with the “dtifit” command, and the diffusion tensors were fitted with a weighted least squares method. A study-specific and age-appropriate FA template was generated from the average of the normalized FA map. A white matter skeleton was calculated and thresholded at a FA of 0.15. We calculated statistics voxel-wise with the randomize program to test for group-level differences in FA between preterm infants receiving CMT or standard care using a general linear model, including the corrected gestational age at the time of the scan as covariate. A statistical threshold of *p* *<* 0.05 was applied after family-wise error correction for multiple comparisons following threshold-free cluster enhancement (TFCE) (Smith and Nichols, 2009).

#### 2.5.3 Functional MRI preprocessing

Functional MRI processing largely followed the workflow previously reported by Mitra et al. for lagged functional connectivity analysis (Mitra et al., 2014) with only minor modifications implemented for infant MRI data. We used an fMRI processing script developed in-house.

First, fMRI data were cleaned of nuisance signal variability. We created subject-specific masks for calculating the time courses of regressors. Axial, coronal, and sagittal T2 MRI were combined to a single, isotropically scaled 3D T2 image using a workflow described previously (Jakab et al., 2019). This image was used to guide a nonlinear deformation that transformed anatomical priors from the UNC neonatal anatomical atlas (Shi et al., 2011) to the subjects. We calculated whole brain, cortex, ventricle, and extra-axial cerebro-spinal (CSF) space, white matter, and thalamus masks for each subject based on the priors in the UNC atlas. The subject-specific priors were thresholded at 50% probability. Next, temporal noise voxels were calculated by thresholding the temporal standard deviation images at the top first percentile value of their non-zero voxels within the brain.

Motion correction was performed using the “fsl_motion_outlier” command in FSL. The resulting six movement parameters, their first temporal derivative, and the frame-wise displacement time course (FD) were saved as a column vectors. Additionally, as suggested by Mitra et al. (2014), we exported the global signal and its first derivative as nuisance time courses. To clean the fMRI images from non-neuronal sources of signal variability, we retained the residuals after first-level regression comprising nuisance time courses using the 3dT project tool in the AFNI software package (Cox, 1996) We adapted the CompCor (Behzadi et al., 2007) approach to reduce noise, in which the first three principal components (PCs) of the anatomically defined time-courses were entered into the confound regression procedure in addition to the movement and global signal time courses. Nuisance signal regression, spatial smoothing with a 5 mm kernel and temporal band-pass filtering retaining frequencies 0.001–0.1 Hz were carried out in one step with 3dT project. fMRI data were also saved without the band-pass step for further processing with independent component analysis. For group-level statistical analysis, the processed fMRI images and anatomical priors were transformed and resampled to standard, T2-weighted templates using the nonlinear registration method described in this paragraph.

Lagged fMRI analysis was performed using the Matlab implementation of the workflow by Mitra et al. (2014).We calculated the latency structure of the rsfMRI acquired for the study population and used the lag maps describing the delay between the signal time course of the whole thalamus and each voxel of the cortex (thalamocortical lag projection map, TCLP) for further statistical analysis. Further information on the fMRI processing and the procedure for generating the TCLP maps are described in the *Supplementary Information*.

The standardized TCLP images were analyzed for group-level differences. We tested between-group differences with a linear model adjusted for the effect of gestational age at birth and time from birth until MRI. Multiple comparison correction of the regression analysis was performed using TFCE with identical settings in the Randomize program as for the TBSS and further analyses. The analysis was restricted to a mask in template space corresponding to 50% probability of the cortex based on the ALBERT neonatal brain atlas. The mean lag of the voxels showing significant between-group effects was saved, and post hoc correlation analysis was performed to show the dose-dependent effect of CMT.

#### 2.5.4 Functional MR processing: dual regression

Dual regression was used to compare resting-state network activity between CMT and standard-care-treated infants. This method consists of two parts: group-level independent component analysis (ICA) and testing of voxel-wise group differences (Beckmann et al., 2007).

We used the standard space fMRI data after preprocessing without bandpass filtering. fMRI data were temporally concatenated using group ICA implemented in the Melodic tool in FSL (Beckmann and Smith, 2004). The number of dimensions was estimated using the Laplace approximation to the Bayesian evidence of the model order, which yielded 51 components. These group-level independent components (ICs) reflect structured signals that exist simultaneously in the data and comprise neuronal signals of interest as well as ICs emerging from head motion and physiological noise.

Dual regression was used to capture the possible effects of CMT on functional signals (Beckmann et al., 2007). A set of spatial maps from the group ICA were used to generate subject-specific versions of the spatial maps and associated time series. These subject-specific time-courses were normalized to allow testing for shape and amplitude effects (Nickerson et al., 2017). Next, the spatial maps were tested voxel-wise for statistically significant differences between the groups using dual regression's default settings and “randomize” (with parameters identical to lag and TBSS analysis), corrected for gestational age at birth and time from birth until MRI. We used a two-tailed Bonferroni correction to adjust for the number of networks that were judged to be nonartefactual in origin.

#### 2.5.5 Graph theoretical analysis of structural and functional connectivity

Systems- and whole-brain-level effects of CMT on structural and functional brain connectivity were tested by graph-theoretical analysis. Structural and functional connectivity matrices were generated from whole-brain tractography using a workflow that was identical to that of a previous study by our group (Jakab et al., 2019).

Seed points for probabilistic diffusion tractography (Parker et al., 2003) were defined as the voxels with FA ≥ 0.1 within a whole-brain mask. Orientation density functions (ODFs) were estimated using fourth order spherical harmonics and a maximum of two local ODF maxima were set to be detected at each voxel and probability density function (PDF) profile was produced from the local ODF maxima. Fiber tracking was carried out on the voxel-wise PDF profile with the Euler interpolation method using 10 iterations per each seed point in the Camino software package (Parker et al., 2003).Tracing stopped at any voxel whose FA was less than 0.2.

Anatomical parcellation was performed using the neonatal regions of interest (ROI) from the UNC Infant atlas (Shi et al., 2011). This comprises 90 areas that were propagated from the Automated Anatomical Labeling atlas (AAL) (Tzourio-Mazoyer et al., 2002) using the transformation that takes the UNC T2-weighted template to the 3D T2 image space and then DTI/fMRI space. The nomenclature of the atlas labels is given in *Supplementary* Table 4, while the atlas alignment procedure is described in the *Supplementary Information*.

Undirected structural connectivity networks were based on whole-brain probabilistic tractography, with nodes corresponding to the AAL in subject space. In the structural connectivity network, edges were defined using the normalized structural connectivity strength, *SC*, which was calculated by using the number of streamlines connecting ROI pairs, normalized for ROI sizes and distances (*Supplementary Information*). Functional connectivity networks (FC) were constructed using zero-lag cross-correlation between the filtered, confounder-corrected time courses between each of the 90 cortical and subcortical brain areas.

Both structural and functional brain network analysis comprised connection (edge) selection with an iterative thresholding procedure. First, we calculated the network cost for each subject without thresholding (actual network edges/all possible edges). We then selected the lowest possible network cost across the study cohort and used this as an upper value of the proportional threshold procedure (van den Heuvel et al., 2017), defined as T_max_. The structural connectivity (SC) networks were thresholded from a density of 1% to T_max_ with the threshold_proportional command in the Brain Connectivity Toolbox, and the surviving edges were kept for both the FA_mean_ and SC networks.

A multivariate covariance analysis using the mancovan toolbox (Mathworks Inc., Mattick, USA) was performed to test the effects of CMT treatment on these nodal graph theoretical parameters. We included demeaned corrected gestational age and time until MRI as confounders. During the mass-multivariate testing of graph theoretical parameters across network nodes, statistical significance was adjusted for multiple comparisons using the Benjamini–Hochberg procedure, implemented in the FDR toolbox in Matlab R2014. As networks were tested at various cost thresholds, the only results that were accepted were those that survived FDR adjustment and consistently showed the same effect direction (positive or negative group difference) in 95% of all the analyzed network costs. Results

### 2.6 Data availability statement

The authors confirm that the data supporting the findings of this study are available within the article [and/or] its supplementary materials. Raw data that support the findings of the study are available from the corresponding author (FBH) upon reasonable request.

## 3 Results

### 3.1 Population

**Fig. 2:**
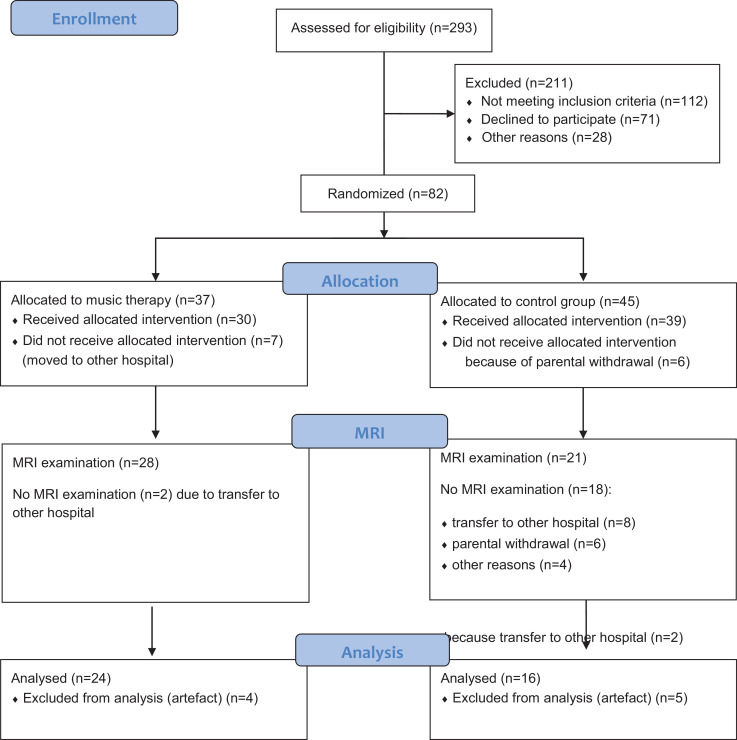
*Flowchart***.** Overview of participant numbers of study enrollment, randomization, allocation, MRI exam and analyses.

Eighty-two preterm infants were recruited (January 2015 -December 2017) and included during the study period between 23 and 31 weeks of gestational age (mean (SD) gestational age 27.99 (± 2.08) at birth) (Fig. 2). After 32 infants were excluded due to relocation to other hospitals and parental withdrawal in the control group (Fig. 2), MRI data were acquired in 49 infants (May 2015-April 2018). Nine infants had to be excluded due to movement artefacts; hence, 40 infants remained as final cohort for the MRI analysis (Fig. 2). In this final cohort, demographic and perinatal variables did not differ between the CMT group (*n* = 24) and the control group (*n* = 16) (*Suppl.* Table 1). These variables did not differ between the MRI cohorts and the infants without MRI either (*Suppl.* Table 2). The final MRI analysis group (*n* = 40) had to be subdivided to sub-cohorts due to the facts that not all imaging sequences were usable for all subjects. For the imaging analysis we formed a diffusion MRI group (*n* = 40, identical to the final MRI analysis group), fMRI group (*n* = 34) and diffusion MRI + fMRI group (*n* = 31). The composition of these groups is detailed in *Suppl.* Table 3.

### 3.2 Feasibility

The recruitment time was longer than anticipated: 2 years instead of 18 months. One reason for this was that recruiting and gaining consent from parents encountered moderate parental rejection (*n* = 24%). Rejection was motivated by two main concerns: firstly, the MRI exam could be an additional stressful event for their infant, and secondly, their infant could be assigned to the control group without CMT (*Suppl.*Fig. 1). However, we are able to recruit more infants than originally intended because the high drop-out rate for the MRI became apparent during the initial recruitment period (Haslbeck et al., 2017).

The drop-out-rate of the study was high, with considerably higher parental withdrawal from the control group than from the music group. Of the 82 randomized preterm infants, not all could receive the allocated intervention, since some study participants were transferred to other hospitals (CMT *n* = 7), and some parents of infants in the control group withdrew informed consent (CG = 6) (Fig. 2). However, no control infants had to be withdrawn from analysis due to contaminating musical stimulation during admission (cf. 31).

The drop-out rate at the time of the MRI exam was 40% (in CMT group 24%, in control group 46%). Only two infants of the 24 in the CMT group dropped out solely due to early transfer to other hospitals. In the control group, 18 infants were lost to the MRI (transfer to another hospital *n* = 8; parental withdrawal of consent for MRI *n* = 6; relocation to another country before term-equivalent age *n* = 3; parental unavailability *n* = 1) (*Suppl.*Fig. 1).

The results demonstrate differing protocol adherence between the two treatment groups, with total protocol adherence rates (CMT group = 100%) and solely external reasons for drop-outs in the CMT group (*Suppl.*Fig. 1). In contrast, the control group demonstrated protocol adherence of only 73% with parental withdrawal (CG=27%) equally affected by parental retention (*n* = 12) and externally determined reasons (*n* = 12) (*Suppl.*Fig. 1)

### 3.3 Intervention sessions

Each infant in the CMT group received the planned CMT sessions (median 15; range: 8–30 sessions; total CMT sessions: 446) during hospitalization (median 5 weeks; range: 3–10 weeks). On average, nine CMT sessions were conducted during kangaroo-care (periods in which the infant is placed on the parents` chest) together with the parents (range: 4–22; total sessions: 262) and six CMT sessions at the bedside with the infant alone (range 2–18; total sessions: 173) (Table 1). No adverse reactions were observed during and directly after the CMT.

**Table 1:** Number of CMT sessions in kangaroo-care, at the bedside and in total.

Characteristic	Total amount	Median	Range
CMT sessions total	446	14.87	8–13
CMT in kangaroo-care with parents	226	9.03	4–22
CMT at bedside with infant alone	173	5.96	2–18

#### 3.3.1 Effects of CMT: tract-based spatial statistics

There were no voxels in which fractional anisotropy (FA) was significantly higher in very preterm infants undergoing CMT than those in controls after correcting with threshold-free cluster enhancement (TFCE) *p* < 0.05. To show possible trends in increased or decreased white matter FA after TFCE, we repeated the analysis with uncorrected p-values, with the threshold for significance set to *p* < 0.01. Three larger clusters of white matter had higher FA in the CMT group, located in the left inferior fronto-occipital fascicle, left precentral gyrus, and left planum temporale (*Suppl.*Fig. 2). The average FA in the significant voxels was 0.149 ± 0.01 in the CMT group and 0.131 ± 0.0083 in the control group.

#### 3.3.2 Effects of CMT: thalamocortical functional connectivity lag

**Fig. 3:**
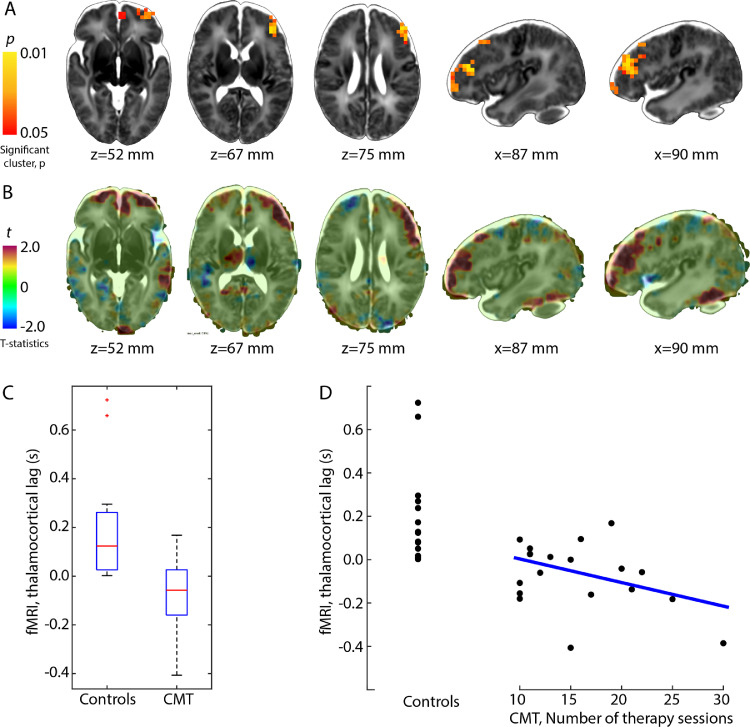
*Effect of creative music therapy on thalamocortical lag structure of resting-state fMRI.*(A) red-yellow overlay: significant between-group differences in the thalamocortical delay, (B) t-statistics map overlaid with the T2-weighted neonatal template. (C) box-plots showing the group effect, (D) scatter plot showing the “dose-dependent” effect of CMT on thalamocortical lag. Linear regression line is fitted on the observations of the CMT cohort only. (For interpretation of the references to color in this figure legend, the reader is referred to the web version of this article.)

Thalamocortical lag was significantly lower in the CMT-treated infants (mean difference: 265 ms, TFCE-corrected *p* < 0.05, see Fig. 3). The between-group effect was localized to parts of the left prefrontal cortex, extending to orbitofrontal regions and anterior parts of the left supplementary motor area (Fig. 3/A). The number of CMT sessions appeared to have a dose-dependent effect and correlated moderately with the thalamocortical lag (product moment correlation coefficient, PMCC, *r* = –0.39). Further analysis of subsignificant results on the t-statistics maps (Fig. 3/B) suggests that the effect may be bilateral, with only the left side reaching conservative significance levels (TFCE-corrected *p* < 0.05). In these maps, the left superior temporal lobe and inferior temporal gyrus were also affected by CMT.

#### 3.3.3 Effects of CMT on functional connectivity differences: dual regression results

**Fig. 4:**
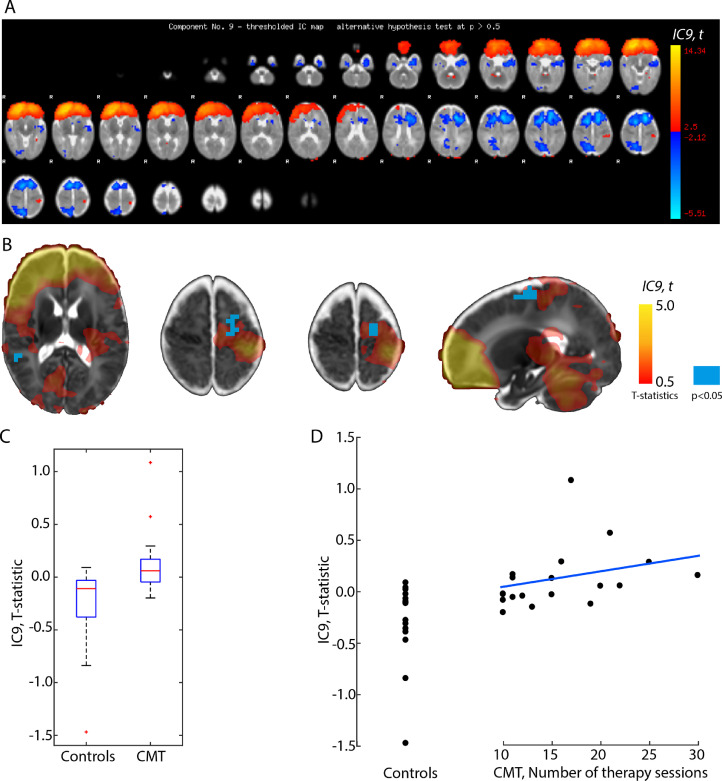
*Effect of creative music therapy on functional connectivity networks.*(A) spatial map showing the distribution of independent network component IC9 (overlay: t-statistics during ICA, thresholded < 2.5 and >−2.12), (B) red-yellow overlay: independent network component IC9, blue overlay: significant between-group effect, *p* < 0.05 (dual regression), (C) box-plots showing the group effect, (D) scatter plot showing the “dose-dependent” effect of CMT on IC strength or extent. Linear regression line is fitted on the observations of the CMT cohort only. (For interpretation of the references to color in this figure legend, the reader is referred to the web version of this article.)

Group-level independent component analysis (ICA) revealed 18 independent components (ICs). Our further analysis focused on the ICs that may overlap with the significant clusters found in the lagged fMRI analysis in frontal, premotor, and supplementary motor regions, and that are not of artefactual origin. We found that IC9 (5.49% of explained variance, 4.12% of total variance), corresponding to bilateral frontal and unilateral postcentral activations (Fig. 4/A), showed significantly higher functional connectivity in the CMT group than in the control group.

The clusters where the amplitude or extent of the IC showed significant between-group effect were located in the left precentral gyrus and partly the left supplementary motor area (Fig. 4/B, blue pixels). The number of CMT sessions appeared to have a dose-dependent effect and correlated moderately with the thalamocortical lag (PMCC, *r* = 0.35, Fig. 4/D).

#### 3.3.4 Effects of CMT: graph theoretical analysis of structural and functional brain connectivity

**Fig. 5:**
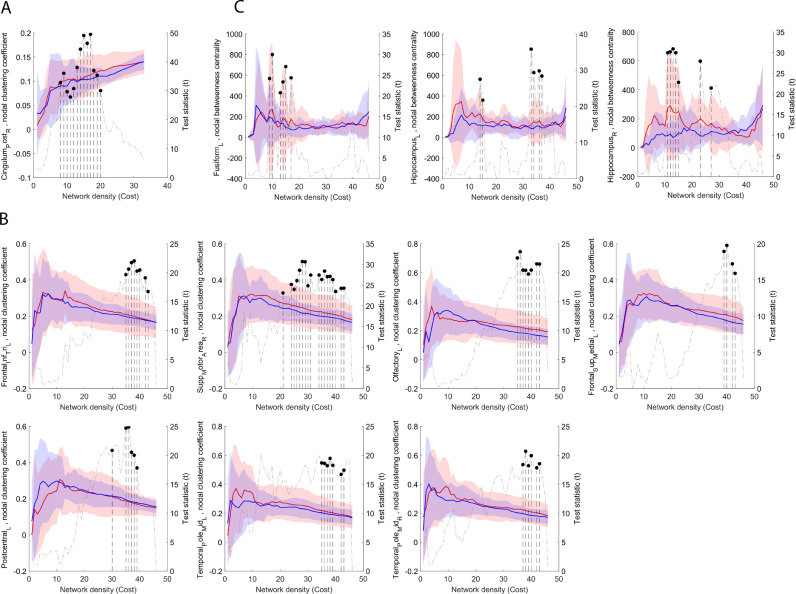
*Effects of CMT on the nodal graph theory characteristics of structural and functional connectivity networks*. (A) effects of CMT on the DTI structural connectivity network, (B) effects of CMT on the clustering coefficient of the functional connectivity network, (C) effects of CMT on the betweenness centrality of the functional connectivity network. In each sub-plot, nodal characteristics are plotted (left axis) as a function of network density, while the right axis shows the T statistic of the multivariate analysis of group differences. Network costs at which significant CMT-control differences appear are marked with segmented line and dot (*p* < 0.05, FDR-corrected across network nodes). Blue lines: mean values, control group, red lines: mean values, CMT group, transparent area reflects the variability of measurements (± 1SD range). The right panels in each sub-plot shows the CGA dependency of the main effect. (For interpretation of the references to color in this figure legend, the reader is referred to the web version of this article.)

The structural connectivity network appears to be largely unaffected by CMT treatment. We only found the clustering coefficient of the right posterior cingulate gyrus to be increased in the CMT group between network densities of 8 and 20% (Fig. 5/A).

In contrast, the nodal-graph theoretical analysis of the functional connectivity network revealed a larger effect of increased clustering coefficient and centrality, indicating more efficient integration. The clustering coefficient increased significantly in the CMT group in six brain regions: left inferior frontal lobe, pars triangularis, left olfactory (orbitofrontal) gyrus, left superior frontal gyrus, medial part, right supplementary motor area, and left and right medial temporal pole. Betweenness centrality was increased in the CMT group in three brain regions: left fusiform gyrus (temporal part) and the left and right hippocampus (Fig. 5/C). The clustering coefficient was decreased in the left postcentral gyrus (Fig. 5/B).

## 4 Discussion

This first randomized controlled pilot trial on CMT shows that is it feasible to implement CMT in a randomized controlled trial (RCT), and more importantly, we provide evidence that CMT has a beneficial effect on functional brain connectivity in very preterm infants.

The successful implementation of the protocol demonstrated its feasibility, although recruiting and consenting the parents revealed moderate parental rejection. Unsurprisingly, this parental rejection was driven by the control group, from which the parents either withdrew consent once allocation was communicated or did not participate in neuroimaging. This information is essential for planning a large RCT, and the high drop-out rate due to transfer to other hospitals for further care needs to be considered when calculating the sample size. The adherence of the control group to the protocol can be improved in future trials by strengthening engagement with and care for the parents in the control group (e.g., with the European Foundation for the Care of the Newborn Infant: EFCNI). We are now involving parents in the conception and planning of a multi-center trial and designing incentives for the control group (European Foundation for the Care of Newborn Infants, 2017).

Our DTI data, when corrected for gestational age at the time of MRI, indicate that structural brain connectivity and white matter microstructure appear to be largely unaffected by CMT. In contrast, increased clustering coefficient and centrality measures for functional brain activity and connectivity, measured in task-negative conditions (rsfMRI), show possible early beneficial effects of CMT in lower thalamocortical processing delay, stronger functional networks, and higher functional integration in predominantly left prefrontal, supplementary motor, and inferior temporal brain regions.

These imply a spatially limited functional improvement of thalamocortical connectivity after CMT without underlying structural changes. While to the best of our knowledge no previously published works have demonstrated the lag structure of intrinsic functional brain connectivity in very preterm infants, decreased thalamocortical lag may indicate the improvement of thalamocortical processing, which is commonly reported to be affected in preterm infants (Ball et al., 2015). Initial evidence suggests that the thalamocortical network reorganizes and improves through long-term musical training because thalamocortical mechanisms are enhanced by integrating of musical tone and rhythm during music processing (Tanaka and Kirino, 2017; Musacchia et al., 2014), which could in part explain our results.

We found enhanced thalamocortical synchronization localized to the temporal lobe and prefrontal cortex and extending to orbitofrontal regions and anterior parts of the left supplementary motor area. We speculate that rhythmic entrainment in CMT may facilitate not only synchronization of breathing, mimicry, and gesture in preterm infants, as demonstrated in a micro-video analysis study (Haslbeck, 2014), but possibly also synchronization at the neural level (Nozaradan, 2014). In a previous study, higher resting-state functional connectivity in auditory, sensory motor, and superior frontal area networks were revealed to be the main effects of exposure to recorded instrumental music (Lordier, 2019). This finding is consistent with our own results and indicates that music processing may affect widespread brain networks early in development and may also affect higher cognitive networks.

We investigated functional connectivity with rsfMRI of the whole brain at term-equivalent age after a period of CMT, a therapeutic intervention with individualized, family-integrating, responsive live music over the whole course of the NICU stay. Interestingly, we observed increased connectivity not only between primary auditory cortices and thalamus, as suggested by Lordier (2019) and Lordier et al. (2019), but also between thalamic, prefrontal, and orbitofrontal regions, which are known to be associated with affective and emotional processing (Koelsch, 2014; Olson et al., 2007), and in parts of the supplementary motor area. The latter is associated with motor initiation, behavioral planning, and word production (Hiroshima et al., 2014; Alario et al., 2006).

Increased connectivity in the prefrontal cortex has been found after or during listening to music in adults (Salimpoor et al., 2013; Lesiuk et al., 2018) and enhanced structural connectivity after intensive musical training in children (Habibi et al., 2016), which is in line with anatomical localization of our results. In general, increased connectivity in the prefrontal cortex is associated with higher-order cognitive functions that are often impaired in preterm infants (Wehrle et al., 2016) such as inhibition, working memory, cognitive flexibility, and planning (Aarnoudse-Moens et al., 2009). Consequently, it will be of interest to assess whether the beneficial mid-term effects of CMT on connectivity in prefrontal regions correlate with long-term executive functions in these infants.

Improved functional connectivity in supplementary motor regions linked to motor planning is currently only associated with extensive musical training over years and not merely listening to music (Tanaka and Kirino, 2017). This observation may lead to the assumption that the preterm infants in the CMT group did not simply listen to the music but instead were actively involved in it, moving along with the music with minimal finger and facial movements, as described in the micro-video analysis of CMT (Haslbeck, 2014). We speculate that interpersonal action coordination may promote functional connectivity in brain regions associated with motor planning and initiation, since it has been argued that these initiating movements to music require the activation of supplementary motor areas (Lima et al., 2016).

Sharda et al. (2018) showed that improvised music therapy may improve functional connectivity between auditory and subcortical regions and between auditory and frontal motor regions in children with autism, which in turn correlate with enhanced social communication (Sharda et al., 2018). Our results suggest to evaluate whether our mid-term findings correlate with long-term communication skills (Haslbeck et al., 2017).

The neuroanatomical and physiological background for the beneficial effect of music training and music therapy remains poorly understood. There is convincing evidence that the development of cerebral connectivity is impaired after preterm birth, and at least part of this impaired connectivity may be attributed to pathological external stimuli. While intrinsic processes induce the formation of an initial connectivity blueprint, extrinsic processes affect its maturation through sensory inputs to the cortex (O'Leary et al., 1992). Prenatal and early postnatal exposure to stimuli, such as music and speech, likely induce changes to the brain's connectivity structure (Partanen et al., 2013), which may steer impaired connectivity development onto a more beneficial trajectory.

Decreased thalamocortical processing delay may indicate more efficient synchrony of intrinsic activity of cortical regions that is relayed through the thalamus. Synchrony with music is a universal human behavior, and extensive brain networks are involved during entrainment to music (Nozaradan, 2014). Recent research has reported groups of nonsensory neurons to be entrained to the beat of music and to synchronize their firing to it in cortical phase entrainment of delta–theta oscillations (Baron-Cohen et al., 2001). This cortical entrainment may also be relevant for music processing and was stronger in musicians with years of musical practice than in nonmusicians (Baron-Cohen et al., 2001). Baron-Cohen et al. (2001) imply that neural entrainment may facilitate music processing and thereby enhance the processing of auditory stimuli in general (Baron-Cohen et al., 2001).

In addition, we found improved structural connectivity of the left posterior cingulate gyrus, associated with attentional focus and empathy (Leech and Sharp, 2014), and increased connectivity between centrality in the left and right hippocampus, associated with the processing of social emotions and inducing appropriate emotional reactions (Immordino-Yang and Singh, 2013). It is argued that the therapeutic response to music with reciprocal action coordination and affective coregulation may facilitate intrabrain coupling and thereby activate brain networks involved in emotion regulation, social behavior, empathy, and cognitive processing (Fachner et al., 2013; Koelsch, 2009).

Our results showed left-hemispheric dominance for the effects of CMT. Increased left-frontal activity is associated with positive emotional experience and a propensity to approach a stimulus whereas increased right-frontal activity is associated with negative emotional experience (Altenmüller et al., 2002). Furthermore, a near-infrared spectroscopy study (Saito et al., 2007) that investigated brain responses of preterm infants to voices indicated that the mothers’ voices only activated the left-frontal area, whereas the nurses’ voices activated the right-frontal area as well. We assume that the processing predominantly identified in the left-frontal area indicates a positive influence of CMT on preterm infants.

Our findings of a dose-dependent effect of CMT are consistent with previous studies. They demonstrate a stronger beneficial effect of music training on functional connectivity with longer intensive training of the sensorimotor network (Li et al., 2018; Luo et al., 2012). However, in the current study, the preterm infants needed only a few weeks of CMT to improve functional connectivity in motor and multi-sensory regions, possibly because CMT is applied during the most plastic period of development. By using this window of opportunity, CMT may provide an effective and possibly neuroprotective and neuroregenerative therapy to support brain development in preterm infants with only a few weeks of exposure. Interestingly, we found a dose-dependent effect of CMT on brain development without an apparent plateau or flattening of the effect, which has important implications for assessing the optimal number and duration of CMT sessions.

CMT is a family-integrating approach aiming at empowering parents in their parental competencies through intuitive vocal interaction with their infant. Reducing parental stress and improving parental self-esteem and responsiveness in music therapy (Haslbeck, 2014; Loewy et al., 2013) may lead to preterm infants being more relaxed and coregulated by the parents. As a result, the infants’ neurological outcomes may improve through a parent–infant interaction effect, as indicated by several studies in family-centered and family-integrated care and in early neonatal developmental intervention programs (O'Brien K et al., 2018). However, while it would be too speculative to conclude at this point that parental involvement enhances functional brain connectivity in preterm infants, future investigation of this avenue would be of value.

We identified factors that limited the data quality and the interpretability of our findings. First, DTI and rsfMRI were only available in a subset of the infants undergoing CMT due to the high drop-out rate; however, this subgroup is representative of the entire study population. Follow-up studies must enroll more infants with good-quality DTI and fMRI to improve statistical power. This is needed to investigate further FA changes within the major white-matter tracts using tract-based spatial statistics, since although our results did not reach conservative significance levels, they may indicate a possible beneficial influence on fractional anisotropy in the left inferior fronto-occipital fascicle, left precentral gyrus, and left planum temporale. Second, all interpretations of improving functional connectivity by CMT are speculative, since the preterm infants’ brain is not a miniature version of the adult brain but a continuously changing system with emerging connectivity. Little is known about how the preterm infant's brain processes music. Thus, further in vivo methods, such as near-infrared spectroscopy, may shed more light on such processing (Saito et al., 2007). Third, no sham intervention was provided. However, since we aimed to examine the potential effect of an additional therapeutic multisensory, family-integrating individualized intervention in neonatal clinical care, a sham procedure was not appropriate. Our study is further limited by the fact that neurobehavioral and neurodevelopmental outcomes have not been reported. It can be assumed that the decreased thalamocortical processing delay in the CMT group will eventually lead to improved cognitive, language, and neurobehavioral outcomes. However, this hypothesis remains to be tested at two and five years (Haslbeck et al., 2017).

To our knowledge, this is the first study investigating the effects of CMT on brain function and structure in preterm infants. A strength of this study is that we evaluated a low-risk, low-cost, family-integrated therapy with the latest MRI analyzing methods incorporating the value of the temporal structure of the brain's spontaneous activity for brain development as well as a whole-brain, network-level analysis (Jakab et al., 2019; Mitra et al., 2014). If we can confirm our positive results of CMT in the planned larger RCT, the findings may have broad clinical implications for preterm infants and their families.

## 5 Conclusion

This trial provides unique evidence that CMT, an individualized, interactive, resource- and needs-oriented approach, has beneficial effects on functional brain activity and connectivity in networks underlying higher-order cognitive, socio-emotional, and motor functions in preterm infants. Future RCTs on CMT should further assess the possible neuroprotective potential of CMT on long-term higher cognitive functions in very preterm infants, on the interaction and bonding of the parents with their child, and importantly also on the well-being of the parents.

## Funding

This work was supported by the Foundation of Anna Müller Grocholski, the Stiftung für Neonatologie, and the Vontobel Foundation. Financial support for A.J. was provided by the OPO Foundation, the Foundation for Research in Science and the Humanities at the University of Zurich, the Forschungszentrum für das Kind Grant (FZK), and the EMDO Foundation, grant number 928. We would like to thank Simon Milligan and Ruth Tuura for her help in language editing the manuscript. C.H. is supported by a SNF PRTC grant 320,030_169,733/2.

## Data availability statement

The authors confirm that the data supporting the findings of this study are available within the article and or its supplementary materials. Raw data that support the findings of the study are available from the corresponding author (FBH) upon reasonable request.

## CRediT authorship contribution statement

**Friederike Barbara Haslbeck:** Conceptualization, Data curation, Formal analysis, Funding acquisition, Investigation, Methodology, Project administration, Resources, Software, Visualization, Writing - original draft. **Andras Jakab:** Formal analysis, Methodology, Resources, Software, Visualization, Writing - original draft. **Ulrike Held:** Supervision, Validation, Writing - review & editing. **Dirk Bassler:** Conceptualization, Supervision, Validation, Writing - review & editing. **Hans-Ulrich Bucher:** Conceptualization, Supervision, Validation, Writing - review & editing. **Cornelia Hagmann:** Visualization, Supervision, Writing - review & editing.

## Declaration of Competing Interest

The authors declare that there are no conflicts of interest regarding the publication of this article.

## Appendix Supplementary materials

Image, application 1

Image, application 2

Image, application 3

Image,4

## Footnotes

2 A single-stringed vibro-acoustic instrument made of wood with 29 strings that are tuned to one base tone with two fifth and three octaves while nevertheless producing many overtones, lacquered with a disinfectable lacquer: http://www.saitenklang.ch/koerpermonochord
