# Supplementary material for: Creative music therapy to promote brain function and brain structure in preterm infants: A randomized controlled pilot study
Source: Neuroimage Clin. 2020 Jan 13;25:102171. doi: 10.1016/j.nicl.2020.102171 (PMC6974781; doi:10.1016/j.nicl.2020.102171)
Supplement: Supplementary file 2 [file mmc2.pdf]

Supplementary Information

1. Generation of a neonatal FA template and transformation of standard space images to DTI and fMRI space

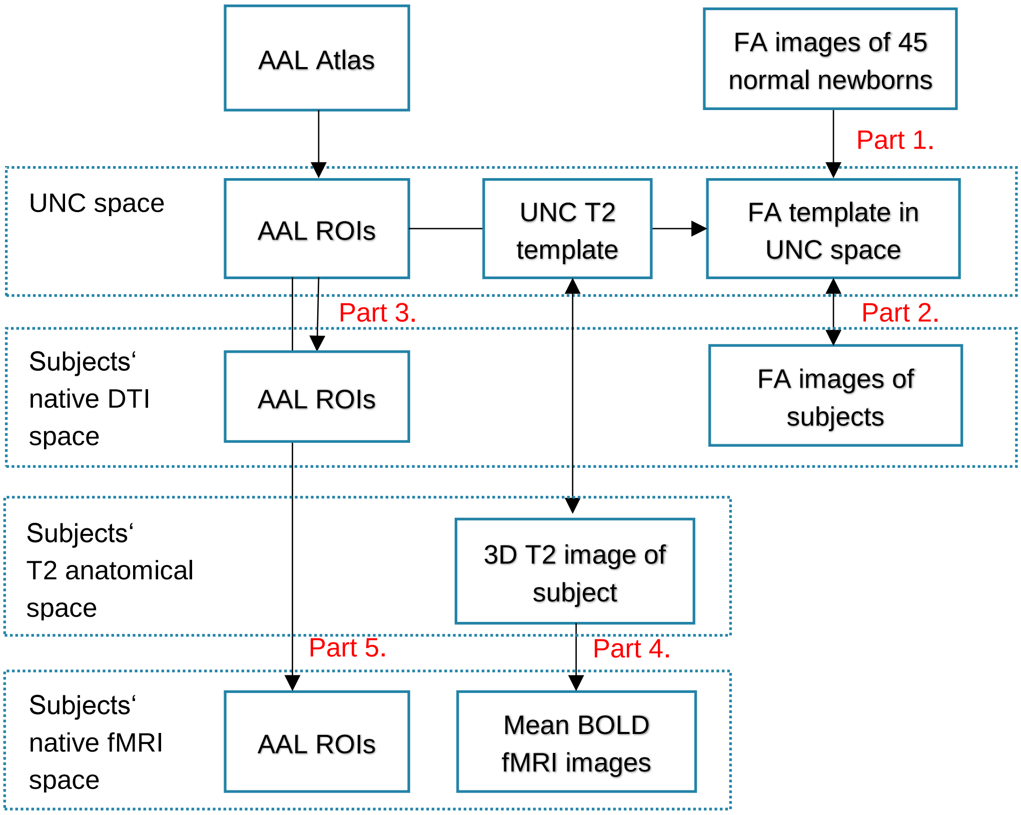

Overview of the template creation and image registration steps in our study.

Part 1: The FA template was constructed in three steps based on DTI data of a separate study population, consisting of 40 normally developing and term-born newborns imaged within 6 weeks after birth (corrected gestational age of the subjects: 42.5 ± 1.9 (39 – 48.7) weeks, 20 females and 20 males). First, we registered the B_0 images of each neonate with the T2-weighted template of the UNC atlas using an affine registration followed by an elastic non-linear registration implemented in the NIFTIreg software (reg_f3d command, control grid size: 9 * 9 * 9 mm, weight of the bending energy penalty term: 0.05, gradient smoothing with a kernel of 4 mm). The transformations arising from this registration step were used to re-sample the Rician filtered FA images of each neonate to UNC template. An initial FA template was then created by averaging these FA images. Next, each subject's FA maps were co-registered to the initial FA template with NIFTIreg with an elastic deformation allowing for finer distortions (reg_f3d command, control grid size: 5 * 5 * 5 mm, weight of the bending energy penalty term: 0.005, gradient smoothing with a kernel of 4 mm).

While tract-based spatial statistics (TBSS) analysis was performed by using a study-specific template created with the standard TBSS pipeline, the structural connectivity analysis required the transformation of AAL ROIs into the subjects' DT-MRI space. In Part 2. of the processing,

the FA template was transformed to each subject's FA image by the reg_f3d command in the Niftireg software applying a fine transformation grid of 8 x 8 x 8 mm. A penalty term was applied with a bending energy setting of 0.0035 to allow for accurate alignments between the template and subject images. The same transformation was used in Part 3. to transform the AAL ROIs to the DTI space of the individual subjects for structural connectivity analysis.

Resting-state fMRI data were normalized to the 3D T2 anatomical scan of the newborns using a 12 degrees-of-freedom linear affine coregistration in FSL (FLIRT), and this linear affine transformation was inverted. The UNC T2 template image was transformed to the 3DT2 image by the reg_f3d command in the Niftireg software applying a fine transformation grid of 8 x 8 x 8 mm (Part 4), and by further transforming this by the previous 12 dof transformation, the UNC-to-BOLD transformation was calculated. This was used during Part 5. To transform the UNC-AAL-ROIs to the subjects' fMRI space for functional connectivity analysis and for calculating anatomical nuisance regressors based on anatomical priors.

2. Lagged functional connectivity analysis

In contrast to standard functional connectivity where exact temporal synchrony is assumed, in lagged fMRI analysis, cross-covariance functions are calculated with a lag term:

$$C_{x_1 x_2}(\tau) = \frac{1}{T}\int x_1\,(t+\tau) * x_2(t)dt$$

where σ_{x1} and σ_{x2} are the temporal standard deviations of signals x_1 and x_2 and T is the interval of integration and τ is the lag (in units of time).

The value of τ at which $C_{x_1 x_2}(\tau)$ exhibits an extremum defines the temporal lag (equivalently, delay) between signals x_1 and x_2. As the absolute value of τ is assumed to be smaller than TR, we calculated extremum of cross-covariance between for all possible pairwise time series using parabolic interpolation, resulting the anti-symmetric matrix TD or time-delay matrix:

$$TD = \begin{bmatrix} \tau_{1,1} & \cdots & \tau_{1,n} \\ \vdots & \ddots & \vdots \\ -\tau_{1,n} & \cdots & \tau_{n,n} \end{bmatrix}$$

To calculate thalamocortical lag projection map (TCLP), we kept the rows of TD that corresponded to the voxels of the thalamus mask in standard space, which resulted in $TD_{thalamus}$. We projected the multivariate data represented in the $TD_{thalamus}$ and matrices onto one-dimensional row vector and mapped this as a 3D image for further statistical analysis, using the technique described by Nikolic and colleagues (Nikolić, 2007). In our case, time projection TCLP meant the mean across the columns of $TD_{thalamus}$:

$$TP = \frac{1}{N}\left[\sum\nolimits_{j=1}^{n} \tau_{1,j} \dots \sum\nolimits_{j=1}^{n} \tau_{n,j}\right]$$

TCLP maps were calculated as unit of TR, which were divided to be in second units. The TCLP maps were standardized to a neonatal brain template corresponding to the 42nd week of gestation.

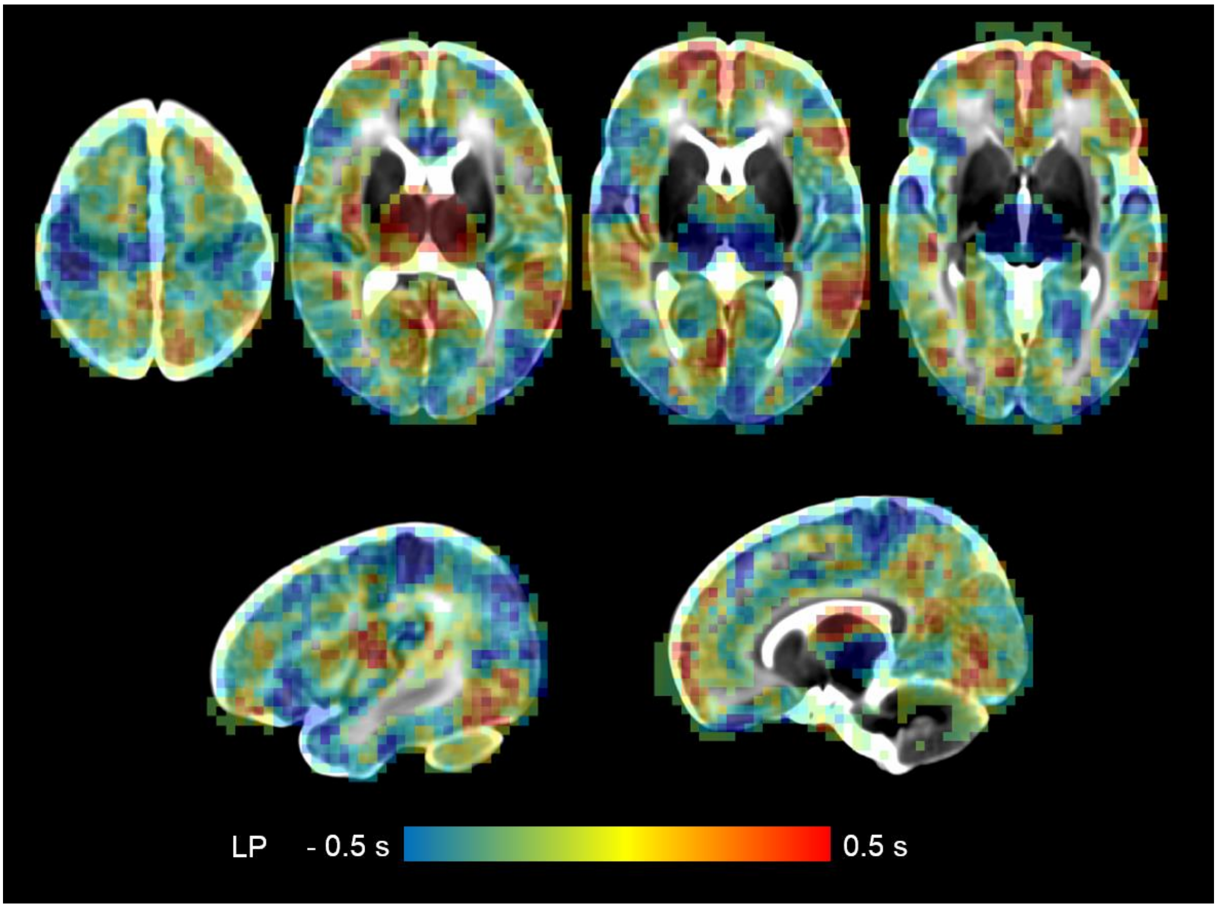

Average lag projection (LP) map over the preterm study cohort. Color code represents LP maps overlaid on top of a T2-weighted neonatal brain template.

Nodal graph theoretical analysis of structural and functional connectivity data

Structural connectivity was represented as undirected structural connectivity networks based on whole-brain probabilistic tractography, with nodes corresponding to the AAL in subject space. We used the following simple model to calculate the structural connectivity strength (SC) between each brain region. Tractography streamline counts connecting any two nodes were normalized for the bias arising from the volumetric differences between the regions of interests corresponding to the nodes, and for the linear bias that arises from more distant brain regions showing more streamlines (Hagmann et al., 2008).

$$SC_{i,j} = \frac{1}{D_{i,j}} * \frac{S_{i,j}}{\frac{V_i + V_j}{2}}$$

Where $SC_{i,j}$ is the structural connectivity strength, $D_{i,j}$ is the Euclidean distance between the node centre-points, $S_{i,j}$ is the number of tractography streamlines connecting nodes i and j, V_i and V_j are the node volumes.

Functional connectivity networks (FC) were constructed using zero-lag cross-correlation between the filtered, confounder corrected time courses between each of the 90 cortical and subcortical brain areas.

For both networks at each cost-thresholded level, the following graph theoretical parameters were calculated using the Brain Connectivity Toolbox for Matlab (Rubinov & Sporns, 2010).

Strength of region *i*:

$$k_i = \sum_{j \in N} w_{ij}$$

where w_{ij} is the connection weight between i and j, N is the entire network.

Betweenness centrality of region *i* (Freeman, 1979):

$$b_i = \frac{1}{(n-1)(n-2)} \sum_{\substack{h,j \in N \\ h \neq j, h \neq i, j \neq i}} \frac{\rho_{hj}(i)}{\rho_{hj}}$$

where ρ_{hj} is the number of shortest paths between h and j, and $\rho_{hj}(i)$ is the number of shortest paths between h and j that pass through i. N are all network nodes. h and j are the neighboring nodes of i.

Weighted local efficiency is the efficiency calculated for a sub-network constructed from the 1st neighbors of the node i:

$$E_{loc}(N) = \frac{1}{n} \sum_{i \in N} \frac{\sum_{j,h \in N, i \neq j} \left(w_{i,j} w_{i,h} [d^w_{i,h}(N_i)]^{-1} \right)^{1/3}}{k_i(k_i - 1)}$$

Where $w_{i,j}$, $w_{i,h}$ are connection weights between nodes i,j and i,h, $d_{i,h}^{w}$ *is the shortest weighted path length between nodes i and h,* k_i *is the degree of node i.*

Weighted clustering coefficient of the network (Onnela et al., 2005; Watts and Strogatz, 1998):

$$C^{w} = \frac{1}{n}\sum_{i\in N} \frac{2t_i^{w}}{k_i(k_i - 1)}$$

where t_i^{w} *is the weighted geometric mean of triangles around node i:*

$$t_i^{w} = \frac{1}{2}\sum_{j,h\in N} (w_{ij}w_{ih}w_{jh})^{1/3}$$
