# Supplementary material for: Creative music therapy to promote brain function and brain structure in preterm infants: A randomized controlled pilot study
Source: Neuroimage Clin. 2020 Jan 13;25:102171. doi: 10.1016/j.nicl.2020.102171 (PMC6974781; doi:10.1016/j.nicl.2020.102171)
Supplement: Supplementary file 3 [file mmc3.docx]

82 randomized 71 declined from 293 = 24% rejection

## Drop-out rates

## exclusion

Drop-out receiving allocated intervention control group

n=6 of 45=13%

Reason: parental withdrawal

Drop-out receiving allocated intervention music group

n=7 of 37=19%

Reason: moved to other hospital

Allocation drop out

n=13 of 82=16%

Drop-out MRI: n=2 of 30=7%

Reason: hospitalization at another hospital at time of MRI exam

Total drop-out music group: n=9 of 37=24%

**→ 100% adherence to protocol**

Drop-out MRI: n=18 of 39=45%

Reasons:

- Externally driven (12): Hospitalization at another hospital (n=8) Mother too ill/ no father (n=1) Moved to another country (n=3)
- Parental withdrawal (6)

Total drop-out control group: n=24 of 45=46%

Reasons:

- 12 externally driven
- 12 parental withdrawal

Wihtdrawal: n=12 of 45=27%

**→ 73% adherence to protocol**

## Attrition:

## Drop-out rates

## Follow-up MRI

MRI exam: n=49

Total drop-out: n=33 of 82=40%

| **Characteristic** | **Reported results** | **MRI Music**  **(n = 24)** ^f^ | **MRI Control (*n* = 16)** ^f^ | ***P*-value** |
| --- | --- | --- | --- | --- |
| Gestational age at birth (weeks) | Mean ± SD | 27.96 ± 2.08 | 27.25 ± 1.85 | .278^b^ |
| Birth weight (g) | Mean ± SD | 1018 ± 283 | 945 ± 324 | .459^b^ |
| Z-score of birth weight | Mean ± SD | -,265 ± ,751 | -,099 ± ,93 | .595^c^ |
| Gestational age at MRI (weeks) | Mean ± SD | 99.46 ± 16.42 | 96.31 ± 16.28 | .555^b^ |
| Birth head circumference (cm) | Mean ± SD | 25.50 ± 2.02 | 24.44 ± 2.31 | .132^b^ |
| Mechanical ventilation days | Mean ± SD | 3.46 ± 5.23 | 2.56 ± 3.35 | .548^b^ |
| Days of Oxygen | Mean ± SD | 47.92 ± 35.12 | 42.63 ± 29.45 | .622^b^ |
| Days of CPAP | Mean ± SD | 30.21 ± 20.30 | 31.56 ± 21.16 | .840^b^ |
| Total parental socio-economic scores (range 2-12) | Mean ± SD | 5.89 ± 2.47 | 6.17 ± 2.92 | .781^b^ |
| Gender (= female) | n (%) | 10 (47.6) | 11 (52.4) | .093^a^ |
| Retinopathy of prematurity | n (%) | 0 (0) | 2 (12.5) | .154^a^ |
| Sepsis^d^ | n (%) | 2 (8.3) | 2 (12.5) | 1.00^a^ |
| Intraventricular haemorrhages (grade 1–2) | n (%) | 2 (8.3) | 4 (25) | .195^a^ |

**Supplementary Table 1: Demographic and clinical parameter comparison of infants included in the MRI analysis with and without music therapy**

Differences of clinical characteristics between groups were assessed with Student’s t-test or Mann-Whitney test as appropriate for the continuous variables and with the chi-square test or the Fisher exact test as appropriate for the categorical variables. SD = standard deviation; CPAP = Continuous Positive Airway Pressure

^a^Chi-square test or Fisher exact test as appropriate.

^b^Student test.

^c^Mann-Whitney test.

^d^ Sepsis proven by positive blood cultures

^e^Oxygen requirement at corrected age of 36 weeks.

^f^ Z-score of birth weight and total parental socio-economic scores (range 2-12) were available for smaller sample sizes: No MRI (n = 17) and MRI (n=30)

No infants in both groups suffered from necrotizing enterocolitis, bronchopulmonary dysplasia or neonatal asphyxia.

| **Characteristic** | **Reported results** | **No MRI (n = 29)** ^f^ | **MRI (*n* = 40)** ^f^ | ***P*-value** |
| --- | --- | --- | --- | --- |
| Gestational age at birth (weeks) | Mean ± SD | 28.07 ± 2.16 | 27.68 ± 2.00 | .432^b^ |
| Birth weight (g) | Mean ± SD | 1108 ± 368 | 989 ± 298 | .143^b^ |
| Z-score of birth weight | Mean rank (sum of ranks) | 28.12 (478) | 21.67 (650) | .121^c^ |
| Birth head circumference (cm) | Mean ± SD | 25.70 ± 2.69 | 25.07 ± 2.18 | .293^b^ |
| Mechanical ventilation days | Mean ± SD | 2.45 ± 3.33 | 3.10 ± 4.54 | .515 ^b^ |
| Days of Oxygen | Mean ± SD | 37.34 ± 29.92 | 45.80 ± 32.68 | .276^b^ |
| Days of CPAP | Mean ± SD | 28.55 ± 16.50 | 30.75 ± 20.39 | .634^b^ |
| Total parental socio-economic scores (range 2-12) | Mean ± SD | 5.41 ± 2.03 | 6.00 ± 2.61 | .428^b^ |
| Gender (= female) | n (%) | 10 (34.5) | 21 (52.5) | .138^a^ |
| Retinopathy of prematurity | n (%) | 2 (6.9) | 3 (7.5) | 1.00^a^ |
| Sepsis^d^ | n (%) | 3 (10.3) | 4 (10.0) | 1.00^a^ |
| Bronchopulmonary disease^e^ | n (%) | 1 (3.4) | 0 (0) | .420^a^ |
| Intraventricular haemorrhages (grade 1–2) | n (%) | 2 (6.9) | 6 (15) | .453^a^ |

**Supplementay Table 2: Demographic and clinical parameter comparison of infants with and without MRI analysis**

Differences of clinical characteristics between groups were assessed with Student’s t-test or Mann-Whitney test as appropriate for the continuous variables and with the chi-square test or the Fisher exact test as appropriate for the categorical variables. SD = standard deviation; CPAP = Continuous Positive Airway Pressure

^a^Chi-square test or Fisher exact test as appropriate.

^b^Student test.

^c^Mann-Whitney test.

^d^ Sepsis proven by positive blood cultures

^e^Oxygen requirement at corrected age of 36 weeks.

^f^ Z-score of birth weight and total parental socio-economic scores (range 2-12) were available for smaller sample sizes: No MRI (n = 17) and MRI (n=30)

No infants in both groups suffered from necrotizing enterocolitis or neonatal asphyxia.
