# Supplementary figures and images for: Creative music therapy to promote brain function and brain structure in preterm infants: A randomized controlled pilot study
Source: Neuroimage Clin. 2020 Jan 13;25:102171. doi: 10.1016/j.nicl.2020.102171 (PMC6974781; doi:10.1016/j.nicl.2020.102171)

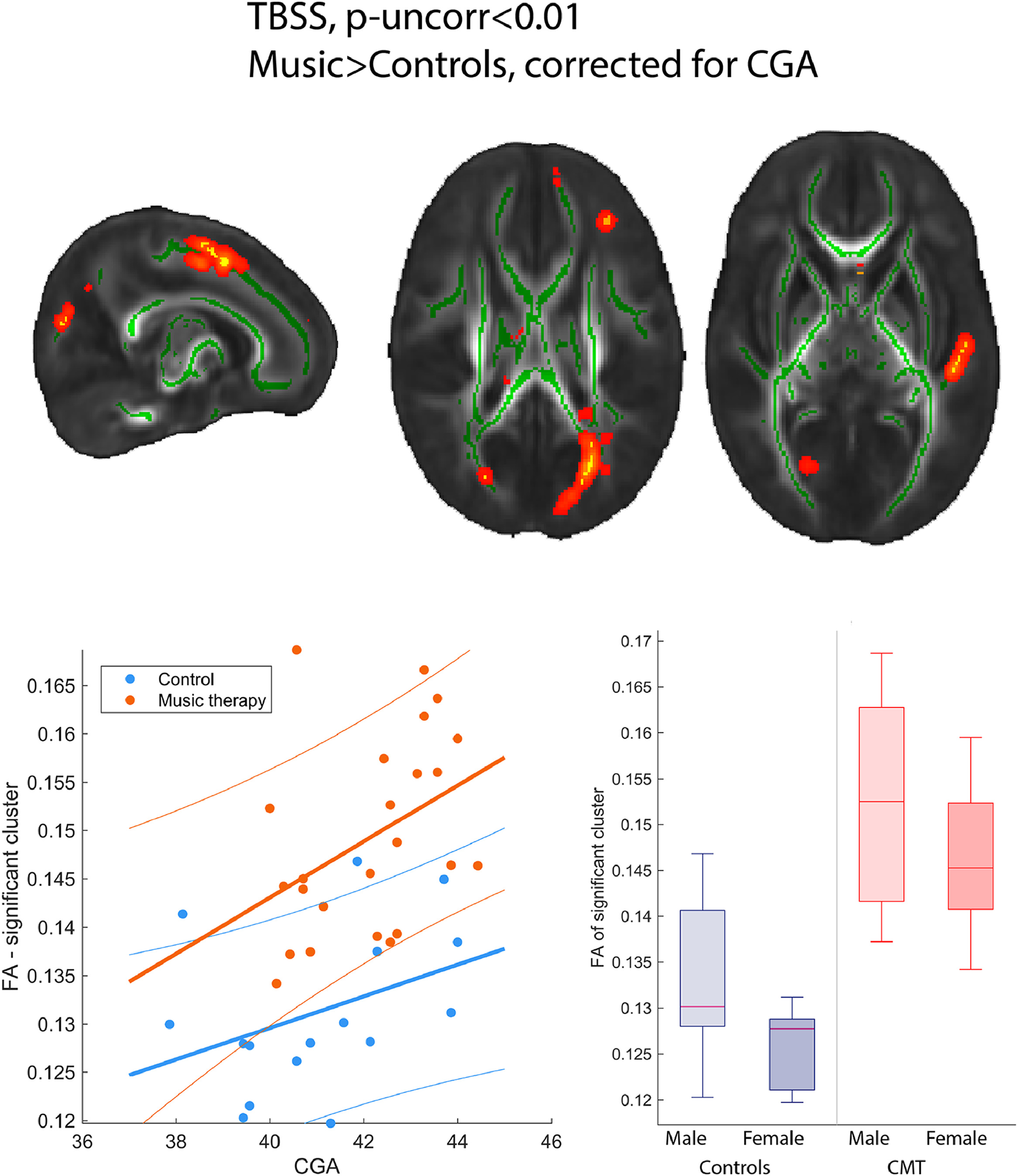

Supplement: Supplementary file 4 [file mmc4.jpg]
